# Development and Validation of an Explainable Machine Learning Model for Major Complications After Cytoreductive Surgery

**DOI:** 10.1001/jamanetworkopen.2022.12930

**Published:** 2022-05-25

**Authors:** Huiyu Deng, Zahra Eftekhari, Cameron Carlin, Jula Veerapong, Keith F. Fournier, Fabian M. Johnston, Sean P. Dineen, Benjamin D. Powers, Ryan Hendrix, Laura A. Lambert, Daniel E. Abbott, Kara Vande Walle, Travis E. Grotz, Sameer H. Patel, Callisia N. Clarke, Charles A. Staley, Sherif Abdel-Misih, Jordan M. Cloyd, Byrne Lee, Yuman Fong, Mustafa Raoof

**Affiliations:** 1 City of Hope National Medical Center, Duarte, California; 2 University of California at San Diego, San Diego; 3 MD Anderson Cancer Center, Houston, Texas; 4 Johns Hopkins University, Baltimore, Maryland; 5 Moffitt Cancer Center, Tampa, Florida; 6 University of Massachusetts, Worcester, Massachusetts; 7 University of Utah, Salt Lake City; 8 University of Wisconsin, Madison; 9 Mayo Clinic, Rochester, Minnesota; 10 University of Cincinnati, Cincinnati, Ohio; 11 Medical College of Wisconsin, Milwaukee, Wisconsin; 12 Emory University, Atlanta, Georgia; 13 Ohio State University, Columbus; 14 Stanford University, Stanford, California

## Abstract

**Importance:**

Cytoreductive surgery (CRS) is one of the most complex operations in surgical oncology with significant morbidity, and improved risk prediction tools are critically needed. Machine learning models can potentially overcome the limitations of traditional multiple logistic regression (MLR) models and provide accurate risk estimates.

**Objective:**

To develop and validate an explainable machine learning model for predicting major postoperative complications in patients undergoing CRS.

**Design, Setting, and Participants:**

This prognostic study used patient data from tertiary care hospitals with expertise in CRS included in the US Hyperthermic Intraperitoneal Chemotherapy Collaborative Database between 1998 and 2018. Information from 147 variables was extracted to predict the risk of a major complication. An ensemble-based machine learning (gradient-boosting) model was optimized on 80% of the sample with subsequent validation on a 20% holdout data set. The machine learning model was compared with traditional MLR models. The artificial intelligence SHAP (Shapley additive explanations) method was used for interpretation of patient- and cohort-level risk estimates and interactions to define novel surgical risk phenotypes. Data were analyzed between November 2019 and August 2021.

**Exposures:**

Cytoreductive surgery.

**Main Outcomes and Measures:**

Area under the receiver operating characteristics (AUROC); area under the precision recall curve (AUPRC).

**Results:**

Data from a total 2372 patients were included in model development (mean age, 55 years [range, 11-95 years]; 1366 [57.6%] women). The optimized machine learning model achieved high discrimination (AUROC: mean cross-validation, 0.75 [range, 0.73-0.81]; test, 0.74) and precision (AUPRC: mean cross-validation, 0.50 [range, 0.46-0.58]; test, 0.42). Compared with the optimized machine learning model, the published MLR model performed worse (test AUROC and AUPRC: 0.54 and 0.18, respectively). Higher volume of estimated blood loss, having pelvic peritonectomy, and longer operative time were the top 3 contributors to the high likelihood of major complications. SHAP dependence plots demonstrated insightful nonlinear interactive associations between predictors and major complications. For instance, high estimated blood loss (ie, above 500 mL) was only detrimental when operative time exceeded 9 hours. Unsupervised clustering of patients based on similarity of sources of risk allowed identification of 6 distinct surgical risk phenotypes.

**Conclusions and Relevance:**

In this prognostic study using data from patients undergoing CRS, an optimized machine learning model demonstrated a superior ability to predict individual- and cohort-level risk of major complications vs traditional methods. Using the SHAP method, 6 distinct surgical phenotypes were identified based on sources of risk of major complications.

## Key Points

**Question:**

Can machine learning provide superior risk prediction compared with the current statistical methods for patients undergoing cytoreductive surgery?

**Findings:**

In this prognostic study, an optimized machine learning model demonstrated superior capability of predicting individual-level risk of major complications after cytoreductive surgery than traditional methods. Cohort-level risk prediction allowed unbiased categorization of patients into 6 distinct surgical risk groups.

**Meaning:**

These results suggest that explainable machine learning methods cannot only provide accurate risk prediction but can also allow identification of potentially modifiable sources of risk on patient and cohort levels.

## Teaser

This prognostic study of US patients who underwent cytoreductive surgery with or without hyperthermic intraperitoneal chemotherapy develops and validates a model for predicting major postoperative complications using machine learning technology.

## Introduction

Risk assessment is fundamental to surgical decision-making. A surgeon must weigh the potential benefits of an operation against both the risks and the alternatives. Such risk assessment can inform decision-making and patient counseling. Importantly, if any modifiable sources of risk are known, it may allow opportunities for intervention aimed at risk reduction prior to surgery. Therefore, there is an unmet need to develop tools that improve surgical risk assessment.

Most surgeons assess risk based on their clinical judgement. While clinical judgement is honed through years of training, interpretation of published data, and patient care experience, there remains an opportunity to augment decision-making through risk prediction tools.^1^ Traditionally, these tools are developed using statistical models, such as linear or logistic regression. However, as the complexity of decision-making increases, these traditional models fall short by failing to account for the complex interactions among predictor variables. This is because traditional models can falsely assume that the variables affecting outcome interact with each other in a linear fashion.^2^ This is often not the case, and the consequences of making this assumption can severely limit the utility of the risk-prediction model.^3^ Alternatively, recent advances in machine learning may be leveraged to develop improved risk prediction tools, especially for high-complexity surgical decisions.^4^

In machine learning, ensemble methods use multiple learning algorithms to obtain better predictive performance than could be obtained from any of the constituent learning algorithms alone. While ensemble machine learning models achieve the goal of higher predictive performance, they are uninterpretable with regards to the sources of risk, limiting their clinical utility.^5^ Recent advances in explainable artificial intelligence methods can now be used to allow interpretation of ensemble-based machine learning models.^6^ These explainable machine learning models hold a great potential in facilitating clinical application of machine learning in the surgical care of patients.

The goal of this study was to develop and validate a novel explainable machine learning model using data from patients undergoing cytoreductive surgery (CRS) with or without hyperthermic intraperitoneal chemotherapy (HIPEC). CRS (with or without HIPEC) is one the most complex operations in surgical oncology, with significant morbidity. Our prior work has demonstrated a significant variation in perioperative care for CRS and HIPEC and patient outcomes.^7^ These observations underscore the need for improved risk assessment to not only improve patient selection but also identify areas of perioperative care optimization.

We developed an explainable machine learning model to (1) accurately predict individual-level risk of major complications; (2) identify global-level clustering of patients in risk categories to understand what we describe as “surgical phenotypes”; and (3) compare the performance of machine learning models to traditional logistic regression models. Lastly, we describe how this explainable machine learning model can potentially be used in the clinic to identify sources of risk and inform surgical decision-making for patients undergoing CRS and HIPEC.

## Methods

### Data Collection

Data for this study was extracted from the US HIPEC Collaborative database, which consists of retrospectively curated data from a multi-institutional cohort of patients who underwent CRS with or without HIPEC. The US HIPEC Collaborative comprises 12 major academic institutions.^8,9^ Each respective institutional review board granted a waiver of informed consent, and data were deidentified. This study followed the Transparent Reporting of a Multivariable Prediction Model for Individual Prognosis or Diagnosis (TRIPOD) reporting guideline for prognostic studies.

### Statistical Analysis

There were a total of 147 predictors (eMethods, eTable 1 in the Supplement). The primary outcome of interest were complications that were graded as 3 or higher based on the Clavien-Dindo classification system.^10^ To predict these surgical complications, we used an ensemble-based machine learning model, a gradient boosting model (hereafter referred to as GBM) as implemented in the lightGBM package (version 2.3.1) in Python.^11^ The training set contained 80% of included patients. The optimized model was then validated by the independent holdout test set, which contained the remaining 20% of the patients (eMethods in the Supplement).

Separately, we developed 2 multivariate logistic regression models to predict surgical complications vs no complications. The first model (hereafter referred to as MLR model 1), which included all significant predictors from univariate logistic regression models and excluded the predictors that were highly correlated with each other. The second model (MLR model 2) used the predictors specified from a previously published paper^12^ that predicted surgical complication. These predictors included Charlson Comorbidity Index (excluding the index malignant neoplasm), symptoms, and prior resection and operative status. We then compared predictive performances of these 2 regression models with that of the GBM.

To facilitate interpretation of the ensemble-based GBM method we used an artificial intelligence SHAP (Shapley additive explanations) method.^13,14^ The SHAP method calculates a total SHAP value for each individual (ie, individual-level total SHAP value), with a higher SHAP value corresponding to a higher likelihood of the target outcome (ie, major complications). To identify surgical phenotypes, patients with similar combinations of individual-level SHAP values were grouped together based on Euclidean distance, an unsupervised distance-based clustering method (eMethods in the Supplement).

## Results

There were a total of 2372 patients in the study cohort. The mean age of the overall cohort was 55 years (range, 11-95 years), and 1366 (57.6%) were women (Table).

**Table.** Selected Baseline and Operative Details

Predictor	Patients, No. (%)
Age, mean (range), y	55 (11-95)
Sex	
Men	1006 (42.4)
Women	1366 (57.6)
Current smoker	156 (6.6)
BMI, median (range)	26.9 (14.4-78.9)
Albumin, median (range), g/dL	4.1 (0.4-7.5)
ASA classification of physical health	
1	10 (0.4)
2	427 (18.0)
3	1617 (68.2)
4	117 (4.9)
Missing	201 (8.5)
Primary site	
Appendiceal	1524 (64.2)
Colorectal	487 (20.5)
Gastric	43 (1.8)
Peritoneal mesothelioma	174 (7.3)
Small bowel	27 (1.1)
Sarcoma	12 (0.5)
Other	99 (4.1)
Missing	6 (0.2)
CEA, mean (range), ng/mL	244.5 (0.2-82 000)
Symptomatic	1256 (53.0)
Prior resection	
None	1787 (75.3)
Yes, without prior CRS and/or HIPEC	409 (17.2)
Yes, with prior CRS and/or HIPEC	176 (7.4)
PCI, mean (range)	14.6 (0-39)
Median operative time (range), h	7.7 (0-20.8)
EBL, median (range)	456.2 (0-7500)
HIPEC	2038 (85.9)
CC score	
0 (No residual tumor)	1319 (55.6)
1 (≤2.5 mm)	539 (22.7)
2 (2.5 mm-2.5 cm)	165 (7.0)
3 (>2.5 cm)	166 (7.0)
Missing	183 (7.7)

Abbreviations: ASA, American Society of Anesthesiology; BMI, body mass index (calculated as weight in kilograms divided by height in meters squared); CC, completeness of cytoreduction; CEA, carcinoembryonic antigen; CRS, cytoreductive surgery; HIPEC, hyperthermic intraperitoneal chemotherapy; PCI, peritoneal cancer index.

### Development of Optimized GBM Model

After splitting the data, there were 1897 patients (80.0%) in the training set, and 475 patients (20.0%) in the test set. Patients with complications grade 3 or higher (ie, major complications) comprised 16% of both the training and the test set. Using the data from the training set, the cross-validation area under the receiver operating characteristics (AUROCs) from the 5 data sets ranged from 0.68 to 0.76 (mean, 0.73), and the cross-validation area under the precision recall curve (AUPRC) ranged from 0.24 to 0.35 (mean, 0.30) (eFigure 1 in the Supplement). The corresponding mean AUROC and AUPRC of the holdout test set were 0.71 and 0.33, respectively.

**Figure 1.**
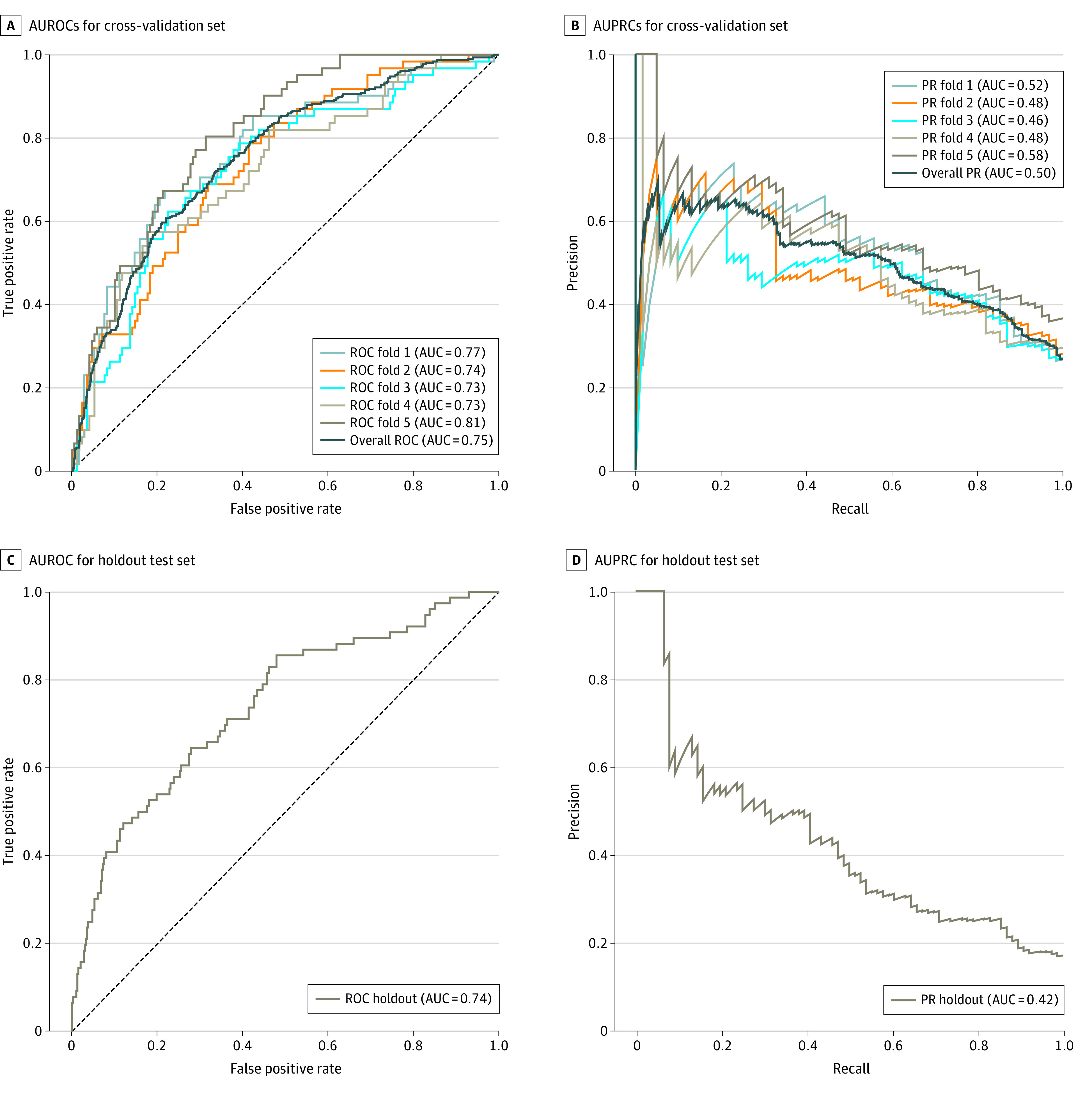
AUROCs and AUPRCs of the Cross-Validation Sets and the Holdout Test Set AUC indicates area under the curve; AUROC, area under the receiver operating characteristics; AUPRC, area under the precision recall curve.

We sought to determine if training the model on patients on the extremes of the outcome (ie, no complication vs complication grade 3 or higher) improved model performance. We reasoned that if the dichotomy was magnified, it would allow for greater accuracy of the optimized GBM model in identifying features of patients with significant complications during training. After excluding patients with grade 1 and 2 complications, there were 1151 patients in the training set; 305 (26.5%) of which had complications that were grade 3 or higher. Indeed, using this subset of the training data, the predictive performances of the model were improved (Figure 1). The mean cross-validated AUROC and AUPRC increased to 0.75 and 0.50, respectively, and the test AUROC and AUPRC of the same holdout test increased 0.74 and 0.42.

### Comparison of Optimized GBM to MLR Models

To evaluate the predictive performance with the optimized GBM, we also developed 2 MLR models using the same subset of the training data (eTable 3 in the Supplement). In MLR model 1, 110 predictors were significantly associated with major complications in the univariate models, and were subsequently used to fit this MLR model. Compared with the optimized GBM, the MLR model 1 had a lower test AUROC (optimized GBM, 0.74 vs MLR model 1, 0.71), test AUPRC (optimized GBM, 0.42 vs MLR model 1, 0.34), test true positive rate (optimized GBM, 0.41 vs MLR model 1, 0.35), and test positive predictive rate (optimized GBM, 0.43 vs MLR model 1, 0.39) (eTable 3 in the Supplement). In the MLR model 2, both CCI and being symptomatic were significant predictors. Compared with the optimized GBM, the MLR model 2 had a much lower test AUROC (MLR model 2, 0.54), test AUPRC (MLR model 2, 0.18), test true positive rate (MLR model 2, 0.38), and test positive predictive rate (MLR model 2, 0.15).

### Individual Risk Prediction Using Optimized GBM

Risk estimates can be extracted from the optimized GBM by estimating SHAP values to allow interpretation of risk on the patient- or global- level. To better interpret the optimized GBM, we first examined the individual-level risk predictions and their sources of risk specified by the SHAP values using the optimized GBM. For the patient with the highest predicted SHAP value (ie, 0.70), high blood loss (1000 mL), longer operative time (9.37 hours), having pelvic peritonectomy and left diaphragm peritonectomy, and not extubated in OR were the sources of risk that led to the high SHAP value (eFigure 2 in the Supplement). On the other hand, the patient with the lowest SHAP value (0.06) had low blood loss (150 mL), shorter operative time (6 hours), a high albumin level (4.3 g/dL), and did not have pelvic peritonectomy or partial colectomy. Moreover, for a patient with a moderate SHAP level (0.21), the predictors that contributed to a higher SHAP value (ie, high white blood cell count [10.9 × 10^3^/μL; to convert to × 10^9^ per liter, multiply by 0.001], low albumin [3.3 g/dL; to convert to gallons per liter, multiply by 10], having pelvic peritonectomy) were offset by the predictors that led to a lower SHAP value (low estimated blood loss [EBL] [225 mL], short operative time [5.4 hours], not having partial colectomy).

### Global Risk Prediction Using Optimized GBM

**Figure 2.**
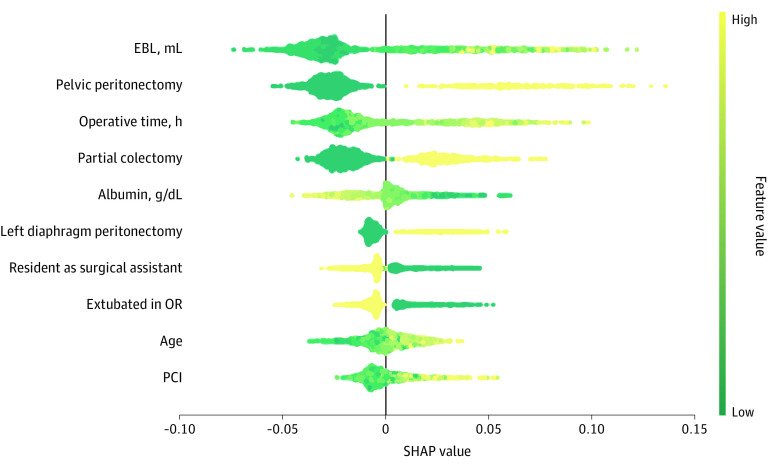
SHAP Summary Plot of the 10 Most Important Predictors For each predictor, the SHAP values for each patient are plotted horizontally, and the magnitude of SHAP values are represented by the x-axis. The importance of the predictors is determined by the predictors’ total SHAP values across all patients (ie, predictor-specific SHAP value). In addition, the values of the predictors are represented by the color: yellow indicates high (or having this condition, if the predictor is binary), green indicates low (or not having this condition). For example, as the EBL increased (from green to yellow), the SHAP values increased, which indicated the likelihood of having complications increased. The binary predictors are pelvic peritonectomy, partial colectomy, left diaphragm peritonectomy, and resident as surgical assistant. EBL indicates estimated blood loss; OR, operative time; PCI, percutaneous coronary intervention; SHAP, Shapley additive explanations. Each point on this plot represents a SHAP value for a predictor and a patient.

We then evaluated the predictors that contributed to the prediction on a global (ie, patient cohort) level. The SHAP summary plot showed that higher volume of EBL, having pelvic peritonectomy and longer operative (OR) time were the top 3 contributors to the high likelihood of complications grade 3 or higher (Figure 2). SHAP values indicated the relative importance of the predictor variable in determining the outcome. Additionally, having partial colectomy, left diaphragm peritonectomy, older age, and higher percutaneous coronary intervention were also associated with a higher likelihood, while higher albumin, having a resident as the surgical assistant, and being extubated in the OR led to a lower likelihood of complications. We then examined the associations between the predictors and the outcome using the 1-way SHAP dependence plots (eFigure 3 in the Supplement). From these plots, we observed the nonlinear associations between EBL and operative time. Specifically, the likelihood of having surgical complications was much higher when EBL was greater than approximately 500 mL and operative time was longer than approximately 9 hours.

### Identification of Surgical Risk Phenotypes and/or Clusters

**Figure 3.**
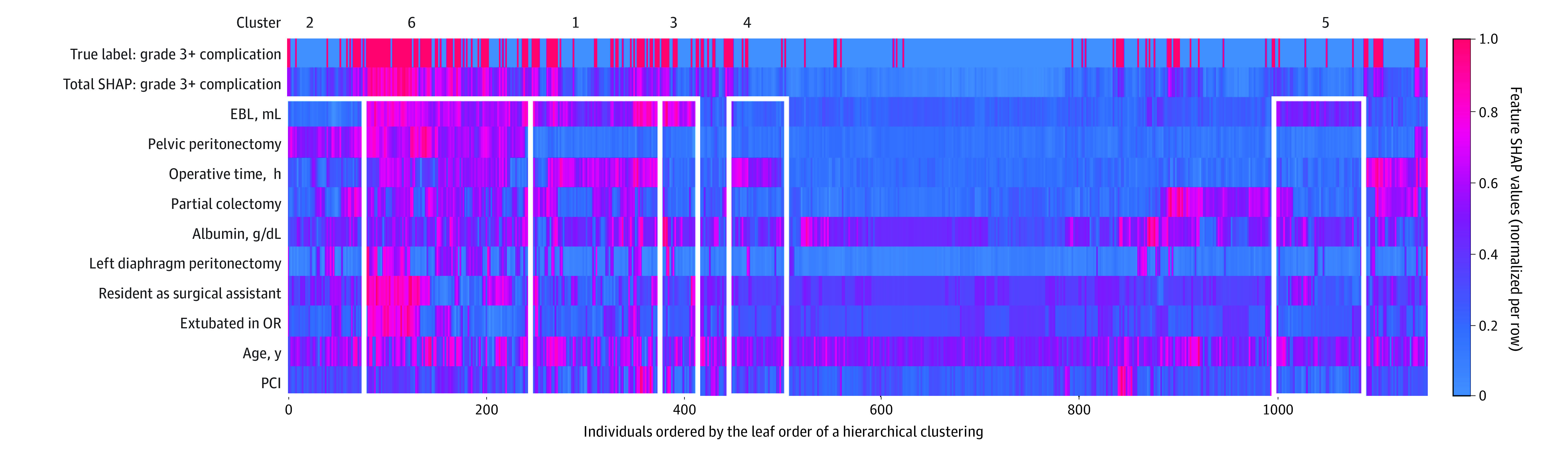
Local Explanation Embedding That Identify Clusters of Patients Who Share Similar Characteristics The local explanation embedding is presented as a heat map of the individual (ie, patient) SHAP values for each of the 10 most important predictors (Figure 2) and is a form of unsupervised distance-based clustering analysis. In this heat map, the patients were grouped based on the “distance” or the similarity between the individual-predictor–specific SHAP values. EBL indicates estimated blood loss; OR, operative time; PCI, percutaneous coronary intervention; SHAP, Shapley additive explanations.

**Figure 4.**
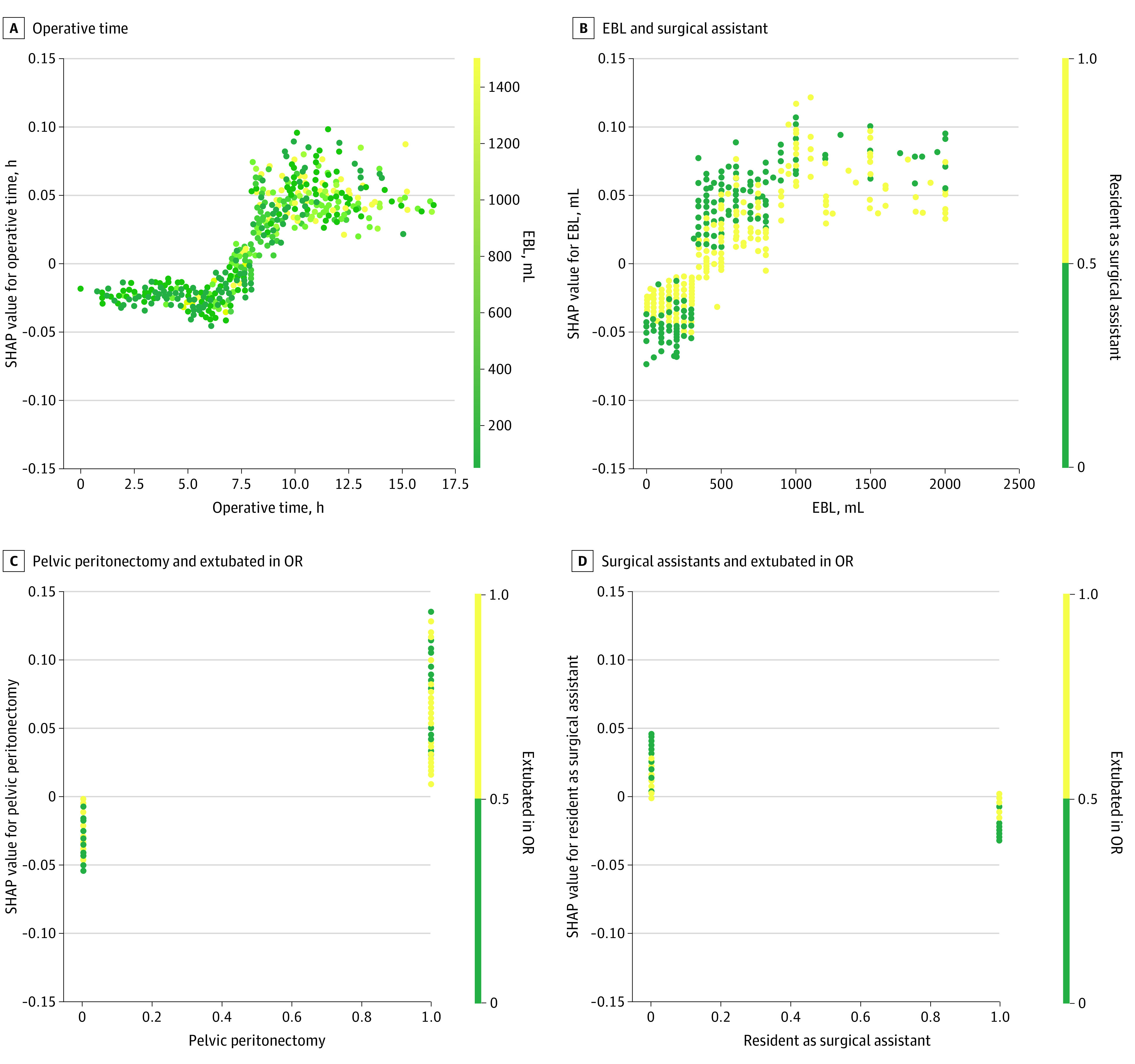
SHAP Dependence Plot of 4 Predictors of Major Complications With CRS CRS indicates cytoreductive surgery; EBL, estimated blood loss; OR, operative time; SHAP, Shapley additive explanations. Values of the first predictor are represented by the x-axis, and the values of the second predictor are represented by the color: yellow indicates high; green indicates low. SHAP values are represented by the y-axis; for categorial variables, a value of 1 indicates yes and 0 indicates no. To interpret these plots, for example: in panel C, patients with pelvic peritonectomy (x-axis value of 1) and not extubated in OR (green color point) were associated with a higher SHAP value, which indicated a higher likelihood of surgical complications.

To identify surgical phenotypes that are associated with complications grade 3 or higher, we generated the plot of local explanation embedding (Figure 3). This plot demonstrated several clusters of patients with similar characteristics that were associated with the likelihood of complications. For simplicity, we first focused on a cluster of higher SHAP values for EBL and OR time, but lower SHAP values for pelvic peritonectomy (cluster 1). This cluster consisted of 145 patients who had total SHAP values that predicted higher likelihood of complications (mean SHAP for patients in cluster 1, 0.44) (eTable 4 in the Supplement). To better understand the relationship between OR time and EBL within this cluster, we evaluated the SHAP dependence plot (eFigure 4 in the Supplement). This plot demonstrated a threshold effect of EBL on OR time—ie, high EBL was only detrimental when the OR time exceeded 9 hours. We then compared this cluster with 4 other clusters (clusters 3 through 5) in which only 1 association of the predictor was present. When patients only had higher SHAP values for EBL (cluster 3 and 5) or OR time (cluster 4), the predicted probability of having complications was not increased (mean SHAP: cluster 3, 0.25; cluster 5, 0.18; cluster 4, 0.24). Similarly, cluster 2, which consisted of patients with high SHAP values for pelvic peritonectomy but low SHAP values for EBL and OR time, had a relatively high likelihood of the outcome (mean SHAP, 0.34). Next, we focused on a cluster of 162 patients with high SHAP values (mean SHAP, 0.68) that included patients with higher SHAP values for EBL, OR time, pelvic peritonectomy, procedure conducted by a surgical assistant in residency, and extubated in OR (cluster 6). From this cluster, we identified 3 interactions and visualized them using the 2-way dependence plots. Specifically, a higher SHAP value or a higher likelihood of complications was associated with (1) more blood loss with the procedure not performed with a resident as the surgical assistant, (2) pelvic peritonectomy and not extubated in OR, and (3) not extubated in OR with the procedure not performed with a resident as the surgical assistant (Figure 4).

## Discussion

In this prognostic study, we developed an ensemble-based machine learning GBM to predict major complications after CRS with or without HIPEC and explored the model’s interpretability using an explainable artificial intelligence SHAP method. This is the first multicenter application of machine learning for risk prediction after CRS and HIPEC.

To our knowledge, machine learning approaches for risk prediction have not been previously evaluated in a complex surgical setting such as CRS. However, prior application of machine learning to less complex surgical risk predictions have produced promising results.^3,15,16,17,18,19^ Extending these findings in a highly complex patient population, this study demonstrated that optimized GBM had an AUROC and AUPRC of 0.74 and 0.42, respectively, which outperformed traditional logistic regression models. This was because the optimized GBM method was able to account for linear and nonlinear interactions among the predictors. In a single institution study, evaluating risk of complications after CRS using logistic regression,^12^ 42 out of 247 patients had grade 3 or greater complications, and the model had an AUROC of 0.67, which was lower than the present study.

The multi-institutional design of the study also provides real-world risk estimation for experienced centers. To ensure that the results were reliable we performed rigorous 5-fold internal validation and a subsequent final validation on a holdout unseen set. Furthermore, we ensured that the holdout set composition was similar to the test cohorts so that the validation was not biased due to cohort composition.

Machine learning models such as the optimized GBM used in this study offer superior risk estimates but are often noninterpretable with respect to the sources of risk. We overcame this issue by visualizing the risk estimates from the optimized GBM model using artificial intelligence–based SHAP values. We identified the top contributors to the risk of surgical complications for individual patients using SHAP force plots as well as for the population using the SHAP summary and dependence plots. As such, this study is one of the most comprehensive clinical applications using the SHAP values. To our knowledge, a few studies have adopted SHAP values to explain their models, but none have included SHAP plots to explain the model both on a local and a global level.^20,21,22,23^ A 2020 study^20^ on predicting acute kidney injury after cardiac surgery used a SHAP summary plot to explain the associations of the top 20 predictors with the outcome on the global level and the nonlinear association of several predictors. Fong et al^21^ used a SHAP dependence plot to explain a 2-way interaction from a model to predict hospital mortality. A third study^22^ also used a SHAP summary plot to explore the top contributing factors that predicted illness severity among COVID-19 patients.

Many of the top predictors of a major complication identified in this study have been previously reported—including EBL, operative time, colon resection, albumin level, and age (Figure 2).^24,25^ As such, these observations provide face validity of the optimized GBM. This study identified additional predictors of major complications, including not being extubated in OR, level of surgical assistance, pelvic peritonectomy, and left diaphragm peritonectomy. Importantly, the large cohort of patients included in this study allowed us to accurately rank the relative importance of these factors.

While our findings provided overall risk estimates for the population, these are unlikely to be useful in the clinical setting. Because of the complex interactions among predictors, factors found to have similar predictive value may have different clinical importance in determining outcome when interacting with each other. A major strength of the SHAP interpretation of the optimized GBM described here is that it provides individual risk estimates (see eFigure 2 in the Supplement for summaries of 3 examples). Annotation of the relative importance of each favorable and adverse predictor could be used by surgical oncologists to counsel patients about important factors driving the risk of a major complication. At the same time, this insight may provide potential avenues for risk modification.

A major limitation of traditional statistical models is that interactions are often ignored or cannot be adequately modeled.^2^ The problem is exacerbated as the complexity of clinical scenarios increases, as with the patient population in the present study. The findings presented here highlight the novel use of GBM to account for nonlinear interactions that are not apparent in traditional models. For instance, 2 of the top individual predictors for developing a significant complication (EBL and OR time) had nonlinear interactions associated with the outcome. By visualizing the SHAP plots of feature importance and dependence we discovered a threshold phenomenon in which the risk of surgical complication increased dramatically only when EBL was high (ie, over 500 mL) and OR time exceeded 9 hours.

This was also the first study, to our knowledge, that used a machine learning–based method to define what we have described as surgical risk phenotypes based on similar sources of risk. For instance, the plot of local explanation embedding enabled us to visualize patient groups with similar characteristics that contributed to a similar likelihood of surgical complications (Figure 3). This is critical not only for patient counseling but also for the development of optimized perioperative recovery pathways that are personalized, yet standardized.^26^ Enhanced recovery after surgery (ERAS) pathways have significantly reduced length of stay and perioperative cost of oncological surgery. There is emerging data that indicates ERAS pathways may have value in planning care for patients undergoing CRS with or without HIPEC. It is also known that these patients represent a heterogenous cohort of patients, and a one-size-fits-all approach to the ERAS pathway is unlikely to be successful. An unsupervised clustering analysis identified 6 distinct clusters with varying predicted risk of a major complications (mean SHAP value range, 0.18-0.68) based on sources of risk, as shown in the plot of local explanation embedding (Figure 3). For practical purposes, these clusters could be distilled to a 3-tier system for implementation of risk-stratified ERAS pathways: low-risk category, consisting of unclustered patients (clusters 2 and 4); intermediate-risk category (clusters 1 and 5); and high-risk category (clusters 3 and 6). The best timing for triaging patients into ERAS pathways may be immediately following surgery, as many of the input variables that pertain to operative details are known. A similar approach to risk-stratified ERAS pathways has been demonstrated to be valuable for pancreatic resections.^26^

### Limitations

This study had several limitations. First, although the optimized GBM model was validated using an unseen holdout set, it would need to be further prospectively validated before widespread adoption. Second, because the model development was based on data from experienced centers, it should be generalized with caution in settings where CRS is not routinely performed. Third, while the model demonstrated moderate discriminative capability, this was far superior to MLR models. Fourth, many of the top predictors were extracted from operative details, which may limit their preoperative use. Almost all patients being considered for CRS and HIPEC are evaluated with contrast-enhanced cross-sectional imaging and/or diagnostic laparoscopy prior to operation. Therefore, surgeons at experienced centers can estimate the likelihood of the extent of resection and infer the input values for the intraoperative variables preoperatively. The optimized GBM model needs to be validated prospectively in this context. Fifth, almost all patients underwent elective curative-intent operations. The findings should be generalized with caution to those undergoing palliative or emergency operations. Finally, adoption of the optimized GBM model in clinical practice as well as integration into electronic medical record will need to be evaluated in future studies.

### Conclusion

The findings of this study demonstrated that an optimized GBM model had a superior predictive performance compared with traditional MLR models for patients undergoing CRS with or without HIPEC. Specifically, we developed and validated an optimized GBM model for accurate risk prediction for a highly complex patient population. Use of SHAP values allowed for interpretability of the optimized GBM, overcoming a major limitation of machine learning models for clinical application. The explanations generated by SHAP plots provide valuable insights into the sources of risk for each individual patient. This information can be used for patient counseling and potential risk modification. We further demonstrated that novel interactions become readily apparent using the SHAP method, which can serve as a valuable research tool for risk mitigation strategies. Finally, on a global level, stratification of patients in different risk categories based on similar sources of risk (what we have described as surgical phenotypes) lays the foundation for future interventional studies focusing on risk-stratified prehabilitation and ERAS pathways.

## Notes

Supplement.
eMethods.
**eFigure 1.** AUROCs and AUPRCs of the Cross-Validation Sets and the Holdout Test Set From the Model Trained With the Entire Training Set **eFigure 2.** Patients With the Highest, Lowest, and Median SHAP Values **eFigure 3.** One-way SHAP Dependence Plot of the Top 10 Most Important Predictors **eTable 1.** List of Variables Incorporated in Risk Models (ie, MLR Model 1) **eTable 2.** Hyperparameters and Their Tuning Range **eTable 3.** Comparison of Predictive Performance Between the GBM and Two Multivariate Logistic Regression (MLR) Models **eTable 4.** Characteristics of the Selected Clusters From the Plot of Local Explanation Embedding (Figure 3)
eReferences.
