# Error in Text

**DOI:** 10.1001/jamanetworkopen.2022.21574

**Published:** 2022-06-22

In the Original Investigation titled “Development and Validation of an Explainable Machine Learning Model for Major Complications After Cytoreductive Surgery,”^1^ published May 25, 2022, the abbreviation PCI was expanded incorrectly. PCI should have been expanded as peritoneal cancer index. This article has been corrected.^1^
