# Supplementary material for: Development and Validation of an Explainable Machine Learning Model for Major Complications After Cytoreductive Surgery
Source: JAMA Netw Open. 2022 May 25;5(5):e2212930. doi: 10.1001/jamanetworkopen.2022.12930 (PMC9133947; doi:10.1001/jamanetworkopen.2022.12930)
Supplement: Supplement. — eMethods. eFigure 1. AUROCs and AUPRCs of the Cross-Validation Sets and the Holdout Test Set From the Model Trained With the Entire Training Set eFigure 2. Patients With the Highest, Lowest, and Median SHAP Values eFigure 3. One-way SHAP Dependence Plot of the Top 10 Most Important Predictors eTable 1. List of Variables Incorporated in Risk Models (ie, MLR Model 1) eTable 2. Hyperparameters and Their Tuning Range eTable 3. Comparison of Predictive Performance Between the GBM and Two Multivariate Logistic Regression (MLR) Models eTable 4. Characteristics of the Selected Clusters From the Plot of Local Explanation Embedding (Figure 3) eReferences. [file jamanetwopen-e2212930-s001.pdf]

Supplemental Online Content

Deng H, Eftekhari Z, Carlin C, et al. Development and validation of an explainable machine learning model for major complications after cytoreductive surgery. *JAMA Netw Open.* 2022;5(5):e2212930. doi:10.1001/jamanetworkopen.2022.12930

eMethods.

eFigure 1. AUROCs and AUPRCs of the Cross-Validation Sets and the Holdout Test Set From the Model Trained With the Entire Training Set

eFigure 2. Patients With the Highest, Lowest, and Median SHAP Values

eFigure 3. One-way SHAP Dependence Plot of the Top 10 Most Important Predictors

eTable 1. List of Variables Incorporated in Risk Models (ie, MLR Model 1)

eTable 2. Hyperparameters and Their Tuning Range

eTable 3. Comparison of Predictive Performance Between the GBM and Two Multivariate Logistic Regression (MLR) Models

eTable 4. Characteristics of the Selected Clusters From the Plot of Local Explanation Embedding (Figure 3)

eReferences.

This supplemental material has been provided by the authors to give readers additional information about their work.

© 2022 Deng H et al. *JAMA Network Open.*

eMethods.

Predictors:
There were a total of 147 predictors, and the complete list of predictors were shown in Table S1. The predictors were from the following groups: Demographic (e.g., Gender, BMI), comorbidity (e.g., Prior cardiac event, diabetes), pre-operative laboratory tests (e.g., Platelet count, WBC count), diagnostic and staging workup (e.g., Extraperitoneal metastases), extent of surgery and operative predictors (e.g., Right diaphragm peritonectomy, extubated in OR), and pre-operative treatment (e.g., type and duration of chemotherapy). To pre-process these predictors, we first grouped them into four data formats – ordinal, binary, categorical, and continuous. We then regrouped the ordinal predictors, such as number of previous CRS, into three categories: None, once; and more than once. In the next step, we pre-processed the missing values. For each of the categorical predictors, a missing category was created. For each of the continuous predictors, the missing values were replaced by its mean or median based on the skewness of its distributions. For example, if the distribution was right or left skewed, the median imputation was applied. If the continuous predictor missed more than 50%, its missing values were replaced by an extreme value (e.g., 999) and a corresponding missing indicator was created. Lastly, we transformed the categorical variables using the one-hot encoder, explicitly creating indicator predictor for each level of the categorical variable.[1]

Predictive modeling
The primary outcome of interest was grade 3 or higher (grade3+) complications based on the Clavien-Dindo classification system.[2] To predict grade 3+ surgical complications, we used an ensemble-based ML model – gradient boosting model (hereafter referred to as GBM), as implemented in the lightGBM package in Python.[3]

GBM is a machine learning method that combines a series of simple tree-based models, such as decision trees.[4] This method first builds a simple tree-based model, and the added more models one at a time to the existing model to minimize the loss function, a function of the difference (aka "the loss) between the prediction of the model and the given outcome value. This process is known as gradient descent. The method is also able reduce overfitting and improve performance by tuning several parameters, such as the number of trees, the fraction subsample, or columns to build each of the tree-based models, and the depth of the trees. The optimal parameters of the final model are selected based on the loss function and the specified evaluation metrics, such as area under the precision/recall curve.

We first trained the model with two different sets of training set. When building a model, data are usually divided into training and test set to avoid overfitting. Based on empirical studies, the best results can be obtained if we use 70-80% of the data for training, and 20-30% data for testing.[5] The first set contained 80% of the patients, which was the entire training set, and the second was a subset of this training set, which only consists of the patients with grade 3+ complications and no complications. We then trained this model on the extremes of outcomes (no complication vs. grade 3+ complication) to improve model performance. We reasoned that if the dichotomy is magnified, it will allow a greater accuracy of optimized GBM model in identifying features of patients with grade 3+ complications during training. Both prediction models were trained using a 5-fold cross validation to find the optimal hyperparameters, and their tuning ranges are listed in

Table S2. During the cross validation, we randomly assigned the patients into five different subsets and ensured that the ratio between the two outcome groups (e.g., grade 3+ versus grade 0) were the same in each of these subsets. We trained the model on four of these groups and tested it on the fifth group. This process was repeated five times so that each patient was used in both training and validation groups. Each time, the areas under the receiver operating characteristic curve (AUROC) and precision-recall curve (AUPRC) were generated, and the optimal values of the hyperparameters that maximized the average AUROC (i.e. overall ROC) was selected. The models with the optimal values of both models were then validated by the independent holdout test set, which contained the remaining 20% of the patients.

Separately, we developed two multivariate logistic regression models to predict surgical complication of grade 3+ vs. no complication. The first model (hereafter referred to as MLR model 1), which included all significant predictors from univariate logistic regression models and excluded the predictors that were highly correlated with each other. The second model - hereafter referred to as MLR model 2 - used the predictors specified from a previously published paper that predicted surgical complication. These predictors included (CCI, excluding the index malignancy), symptoms, and prior resection and operative status.[6] We then compared predictive performances of these two regression models to that of the GBM.

Local and Global Interpretation of ML models

Although ensembled-based GBMs may provide good prediction accuracy, they cannot be applied in the clinic because the generated output cannot be interpreted. To facilitate interpretation of the ensemble-based GBM method we used an artificial intelligence SHAP (SHapley Additive exPlanations) method.[7,8] This SHAP method calculates a total SHAP value for each individual (i.e., individual-level total SHAP value) – a patient with a higher SHAP value corresponds to a higher likelihood of the target outcome i.e. major complications. In addition, the method breaks down the SHAP values of predictors for a given individual to predictor specific SHAP value which examines how the levels of predictors contribute to this individual's total SHAP value. These predictor-level SHAP values can also be aggregated to show how each of the predictor affects the outcome across all individuals. SHAP values enable the interpretation of the model both on a local (e.g., individual prediction) or a global (e.g., population trend) level.

To facilitate the interpretation, the method creates the force, summary, and dependence plots to visualize the local- and global-level SHAP values. On the local level, the force plot shows the individual-level total SHAP value and the predictors that contribute to this value. On the global level, the summary and dependence plots display the predictor specific SHAP value across all individuals. Specifically, the summary plot ranks the predictors' predictive ability to the outcome as well as the direction of the effects; the dependence plots not only display the direction of effects, but also shows the non-linear associations and interactions between predictors. In addition, individual-level predictions can be embedded into an explanation space (i.e., Local explanation embedding) to make the global interpretation. In this space, individuals with a similar combination of individual-level SHAP values were grouped together based on Euclidean distance - an unsupervised distance-based clustering method. These clusters (or patient groupings) allow us to discover common patterns among a subgroup of the population, and to interpret how these patterns together lead to the outcome.

© 2022 Deng H et al. *JAMA Network Open.*

eFigure 1. AUROCs and AUPRCs of the Cross-Validation Sets and the Holdout Test Set From the Model Trained With the Entire Training Set

.

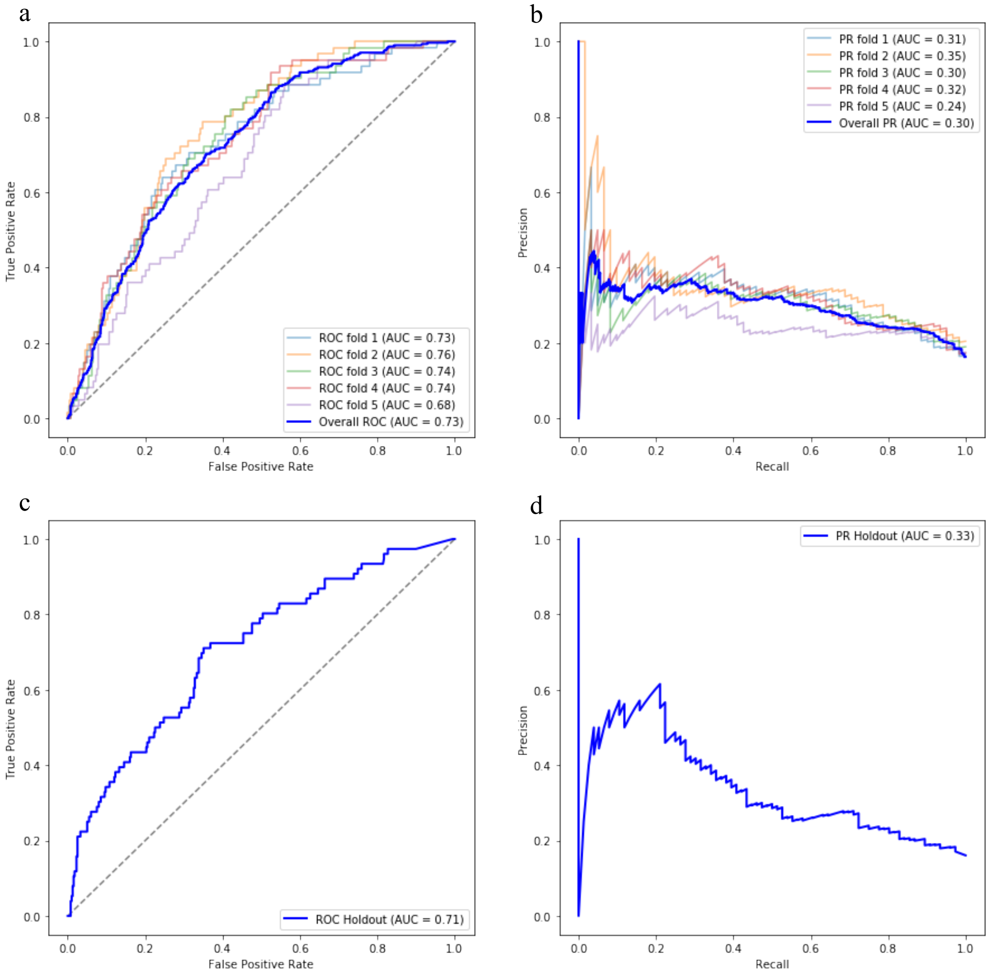

© 2022 Deng H et al. *JAMA Network Open.*

eFigure 2. Patients With the Highest, Lowest, and Median SHAP Values

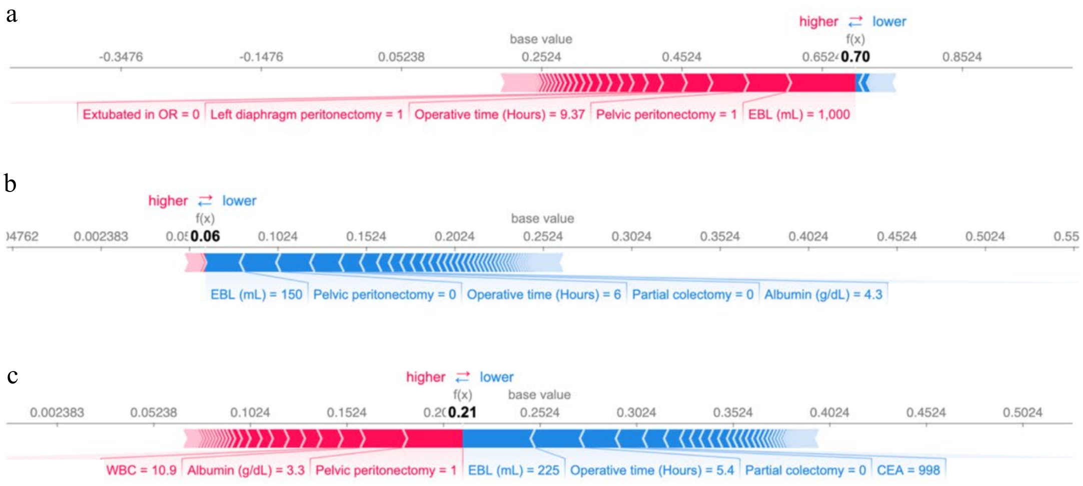

© 2022 Deng H et al. *JAMA Network Open.*

eFigure 3. One-way SHAP Dependence Plot of the Top 10 Most Important Predictors

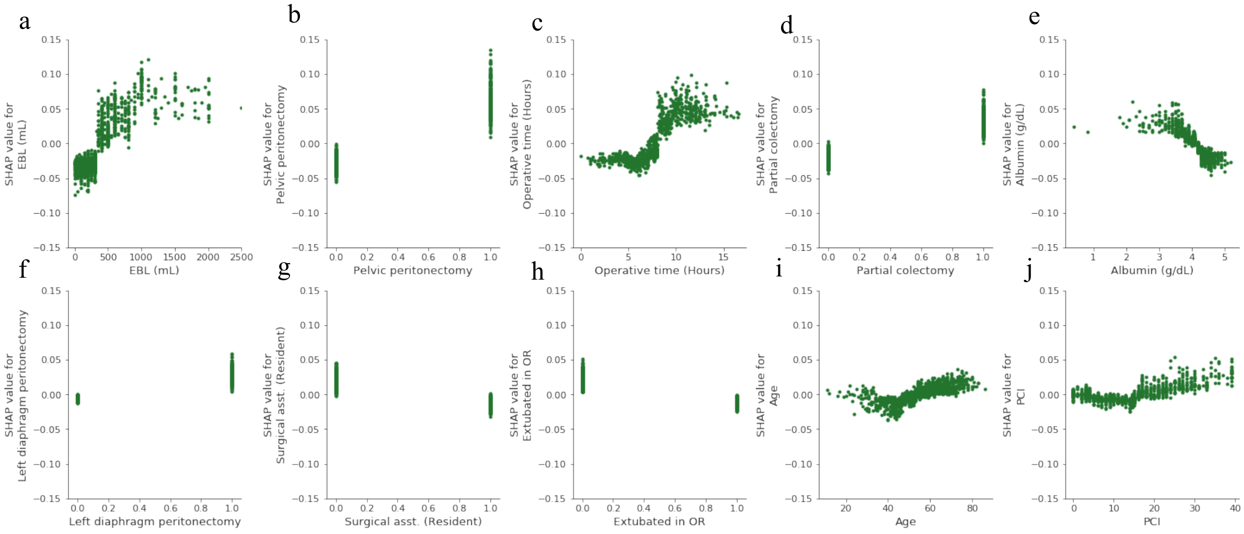

[1] These are the one-way dependence plots, which shows the association between a predictor and the outcome. Specifically, the values of the predictor are represented by the x-axis, and its SHAP values are represented by the y-axis. To interpret these plots, for example, in (a), patients with higher EBL (as x-axis increased) were associated with a higher SHAP value, which indicated a higher likelihood of surgical complications (y-axis also increased).

© 2022 Deng H et al. *JAMA Network Open.*

eTable 1. List of Variables Incorporated in Risk Models (ie, MLR Model 1)

Groups of the predictors	Predictor
Demographic	Hospital, gender, age, race, BMI, health insurance, ASA class, functional status, ECOG performance status, histology
Comorbidity	HTN, diabetes, prior cardiac event, CHF, dyspnea, smoking history, severe COPD, chronic steroids, ascites, disseminated cancer, history of MI, history of PVD, history of CHF, history of CVA, anticoagulant, depression, rheumatic or connective tissue disease, history of PUD, symptomatic, GI bleed, GI obstruction, diarrhea, pain, nausea/vomiting, anorexia, fatigue, anemia, constipation, GERD/dyspepsia, history of prior appendectomy, chronic kidney disease, liver disease, known genetic syndrome, drinker, smoker, previous abdominal surgery, other concurrent malignancy
pre-operative laboratory tests	Platelet count, WBC count, neutrophil, lymphocyte, monocyte, hemoglobin, albumin, preoperative prealbumin level, last bilirubin, creatinine, HbA1C, glucose, CEA, CA19.9, CA.125, C reactive protein
Diagnostic and staging workup	Extraperitoneal disease, liver metastases, lung metastases, retroperitoneal lymph node metastases, other metastases, preoperative TPN
Extent of surgery and operative predictors	Surgeon volume, emergency surgery, wound class, previous HIPEC, number of previous CRS, operative intent, indication for palliative resection, pre admission, reason for pre admission, **ureteral stents, surgical assistant, operative time, PCI, CCR, right diaphragm peritonectomy, major right peritonectomy, prophylactic right chest tube, perfusion of chest, right diaphragm resection, left diaphragm peritonectomy, major left peritonectomy, prophylactic left chest tube, left diaphragm resection, cholecystectomy, porta hepatis, splenectomy, distal pancreatectomy, omentectomy, lesser omentum, right gutter peritonectomy, left gutter peritonectomy, pelvic peritonectomy, hysterectomy, oophorectomy, partial cystectomy, lymphadenectomy, previous CRS, gastrectomy, low anterior resection, diverting loop ileostomy, number of small bowel resections, mesenteric peritonectomy, small bowel tumor excision, end ileostomy colostomy, appendectomy, nephrectomy, ureteral resection, why ureteral resection, liver capsular resection, formal liver resection, caudate resection, intraoperative ablation, pringle used, abdominal wall resection, component separation, same surgeon performed, placement of gastrostomy tube, jejunostomy feeding tube, placement of IP catheter, number of drains, extubated in OR, partial colectomy, small bowel resection, abdominal wall reconstruction, Intra OP drain placement, estimated blood loss**

© 2022 Deng H et al. *JAMA Network Open.*

Pre-operative treatment	IP chemotherapy, targeted temperature, closed vs. open, chemotherapy 1, chemotherapy 1's duration, systemic chemotherapy, perfusion terminated, neoadjuvant chemo regimen, neoadjuvant bevacizumab, neoadjuvant immunotherapy, neoadjuvant targeted kinase inhibitor, neoadjuvant chemotherapy

Bold = Variables derived at the time of surgery

© 2022 Deng H et al. *JAMA Network Open.*

eTable 2. Hyperparameters and Their Tuning Range

Hyperparameter Name	Description	Tuning range
learning_rate	Boosting learning rate.	Fixed at 0.1
Max_delta_step	Limit the max output of tree leaves	Fixed at 1
Scale_pos_weight	Balancing of positive and negative weight.	Fixed at the ratio of two outcome groups
num_boost_round	Number of gradient boosted trees.	Fixed at 1000 for tuning the number of tree, and the optimal number of trees for the final model[1] was determined using 5-fold CV.
max_depth	Maximum tree depth for base learners.	Fixed at 6 for tuning the number of tree, and randomly picked from 3 to 10 in the final model.
min_child_weight	Minimum sum of instance weights needed in a child.	Fixed at 1 for tuning the number of tree, and randomly picked from 1 to 5 in the final model.
colsample_bytree	Subsample ratio of columns when constructing each tree.	Fixed at 1 for tuning the number of tree, and uniformly picked from 0.4 to 0.9 in the final model.
Subsample	Subsample ratio of the training instance.	Uniformly picked from 0.4 to 0.9 in the final model.
Reg_alpha	L1 regularization	Uniformly picked from 10 evenly spaced points between decades 10^-2 and 10^2
Reg_lambda	L2 regularization	Uniformly picked from 10 evenly spaced points between decades 10^-2 and 10^2

[1] A two-stage tuning process was adopted to train the model. In stage I, the initial number of gradient boosted tree (num_boost_round) was tuned in a model with all other parameters fixed at a default value. In stage II, using the number of tree tuned from the stage I model, the other parameters of the models, which included max depth, min child weight, subsample rate, column sample rate, regularized alpha and lambda levels, were further tuned. The model prediction and interpretion were generated using the stage II model with the tuned parameters.

© 2022 Deng H et al. *JAMA Network Open.*

eTable 3. Comparison of Predictive Performance Between the GBM and Two Multivariate Logistic Regression (MLR) Models

	Optimized GBM[1]	MLR model 1[1,2]	MLR model 2[1,3]
AUROC[4]	0.74	0.71	0.54
AUPRC[4]	0.42	0.34	0.18
Threshold[5]	0.41	0.47	0.27
Positive class[6]	76	76	76
True positive	31	30	29
False positive	41	55	166
False negative	45	45	47
True positive rate (TPR)	0.41	0.35	0.38
Positive predictive value (PPV)	0.43	0.39	0.15

[1] All three models were developed with the subset of the training set excluding the patients with grade 1 and 2 complications.

[2] MLR model 1 included all significant predictors from univariate logistic regression models and excluded the predictors that were highly correlated with each other.

[3] MLR model 2 included CCI score, symptomatic, and previous CRS and HIPEC status.

[4] The AUROC and AUPRC of the test set were reported.

[5] The threshold to classify the predicted probability was determined at the recall and precision of 40%.

[6] Positive class was the total number of patients who had the outcome.

© 2022 Deng H et al. *JAMA Network Open.*

eTable 4. Characteristics of the Selected Clusters From the Plot of Local Explanation Embedding (Figure 3)

Clusters	Number of patients	Mean[1]	Standard deviation[1]	Minimum[1]	Maximum[1]
1	145	0.44	0.20	0.06	0.81
2	78	0.34	0.16	0.10	0.77
3	27	0.25	0.12	0.10	0.62
4	55	0.24	0.16	0.05	0.66
5	91	0.18	0.11	0.05	0.64
6	162	0.68	0.18	0.28	1.00

[1] Summary statistics of the total SHAP values for the patients in the cluster.

© 2022 Deng H et al. *JAMA Network Open.*

eReferences

1. McKinney W. Data Structures for Statistical Computing in Python. In: Millman SevdWaJ, ed. *Proceedings of the 9th Python in Science Conference* 2010:51-56.
2. Clavien PA, Barkun J, de Oliveira ML, et al. The Clavien-Dindo classification of surgical complications: five-year experience. *Ann Surg*. Aug 2009;250(2):187-96. doi:10.1097/SLA.0b013e3181b13ca2
3. Ke G, Meng Q, Finley T, et al. LightGBM: A Highly Efficient Gradient Boosting Decision Tree. In: Guyon I, Luxburg UV, Bengio S, et al, eds. *Advances in Neural Information Processing Systems 30*. Curran Associates, Inc.; 2017.
4. Friedman JH. Stochastic gradient boosting. *Computational Statistics & Data Analysis*. 2002/02/28/ 2002;38(4):367-378. doi:https://doi.org/10.1016/S0167-9473(01)00065-2
5. Gholamy A, Kreinovich V, Kosheleva O. Why 70/30 or 80/20 Relation Between Training and Testing Sets: A Pedagogical Explanation. 2018:
6. Baumgartner JM, Kwong TG, Ma GL, Messer K, Kelly KJ, Lowy AM. A Novel Tool for Predicting Major Complications After Cytoreductive Surgery with Hyperthermic Intraperitoneal Chemotherapy. *Ann Surg Oncol*. May 2016;23(5):1609-17. doi:10.1245/s10434-015-5012-3
7. Lundberg SM, Lee S-I. A Unified Approach to Interpreting Model Predictions. In: Guyon I, Luxburg UV, Bengio S, et al, eds. *Advances in Neural Information Processing Systems 30*. Curran Associates, Inc.; 2017:4765--4774.
8. Lundberg SM, Erion GG, Lee S-I. Consistent Individualized Feature Attribution for Tree Ensembles. 2019

© 2022 Deng H et al. *JAMA Network Open.*
